# Association between general anesthesia in early childhood and neurodevelopment up to 4 years of age: the Japan Environment and Children’s Study

**DOI:** 10.1007/s00540-024-03359-9

**Published:** 2024-06-07

**Authors:** Takahisa Nagai, Yoshiko Yoda, Narumi Tokuda, Yasuhiro Takeshima, Munetaka Hirose, Masayuki Shima, Japan Environment, Children’s Study (JECS) Group, Masayuki Shima, Michihiro Kamijima, Shin Yamazaki, Yukihiro Ohya, Reiko Kishi, Nobuo Yaegashi, Koichi Hashimoto, Chisato Mori, Shuichi Ito, Zentaro Yamagata, Hidekuni Inadera, Takeo Nakayama, Tomotaka Sobue, Seiji Kageyama, Narufumi Suganuma, Shoichi Ohga, Takahiko Katoh.

**Affiliations:** 1 Department of Public Health, School of Medicine, Hyogo Medical University, Nishinomiya, 663-8501 Japan; 2 Hyogo Regional Center for the Japan Environment and Children’s Study, Hyogo Medical University, Nishinomiya, 663-8501 Japan; 3 Department of Pediatrics, School of Medicine, Hyogo Medical University, Nishinomiya, 663-8501 Japan; 4 Department of Anesthesiology and Pain Medicine, School of Medicine, Hyogo Medical University, Nishinomiya, 663-8501 Japan; 5 School of Nursing, Hyogo Medical University, 1-3-6 Minatojima, Chuo-Ku, Kobe, Hyogo 650-8530 Japan

**Keywords:** Birth cohort study, Children, General anesthesia, Neurodevelopment, The Ages and Stages Questionnaires, Third Edition (ASQ-3)

## Abstract

**Purpose:**

The effects of general anesthesia on neurodevelopment in children remain controversial. We explored the relationship between general anesthesia and neurodevelopment in children participating in the Japan Environment and Children’s Study (JECS).

**Methods:**

This study enrolled children born between 37 and 41 weeks of pregnancy via single-vaginal delivery to pregnant women registered in the JECS between January 2011 and March 2014. Data were collected from mother-completed questionnaires and medical transcripts. Neurodevelopment in five domains was assessed every 6 months between 12 and 48 months of age, using the Ages and Stages Questionnaires. The associations between general anesthesia exposure during early childhood and neurodevelopment in children were evaluated at each time point. Adjusted odds ratios and 95% confidence intervals were estimated after covariate adjustment using logistic regression models.

**Results:**

Children who received general anesthesia before age 1 year had higher risks of neurodevelopmental delay in all five domains throughout the observational period. The largest risk was for gross motor delay at 18 months (adjusted odds ratio: 3.51; 95% confidence interval: 2.75–4.49). The effects on the incidence of neurodevelopmental delays after age 3 were not observed except for problem solving at 48 months. The risk of neurodevelopmental delay in children who first received general anesthesia after age 1 was considerably small.

**Conclusions:**

This study suggests that general anesthesia administration before age 1 is associated with neurodevelopmental delay during 1–4 years of age. The risk of general anesthesia after age 1 may be small.

## Introduction

Although many studies have discussed and reported the effects of general anesthesia on neurodevelopment in young children, no definitive conclusions have been reached [1–10]. In 2016, the U.S. Food and Drug Administration (FDA) issued a drug safety bulletin warning that multiple procedures or the use of longer than 3 h of general anesthesia and analgesics during surgery or other painful and stressful procedures in children under 3 years of age or during the third trimester of pregnancy may affect brain development in children [11, 12]. This was based on the effect of anesthetics observed in animal studies [13], which demonstrated that anesthetics were associated with neurological damage, such as widespread neuronal and oligodendrocyte cell loss, synaptic morphology, and neurogenesis during the period of rapid brain development. Some anesthetics (e.g., propofol, benzodiazepine, isoflurane, sevoflurane) have been associated with *ɤ*-aminobutyric acid receptor-mediated disorder and others, such as ketamine and nitrous oxide (N_2_O), cause the reduction of rare gliocytes by *N*-methyl-*D*-aspartic acid receptors [5–8]. However, conclusive clinical evidence on the deleterious effect of anesthetics on neurodevelopment is limited.

To date, three large-scale prospective human studies, namely the Pediatric Anesthesia Neurodevelopment Assessment (PANDA) study [14], Mayo Anesthesia Safety in Kids (MASK) study [15, 16], and General Anaesthesia compared to Spinal Anesthesia (GAS) trial [17, 18] reported that a single brief exposure to general anesthesia (median duration was 54 min) did not cause neurodevelopmental deficits in children. However, the effects of prolonged general anesthesia exposures were inconclusive.

Using the data from the Japan Environment and Children’s Study (JECS), a large ongoing birth cohort study in Japan, we previously investigated the effects of general anesthesia administered before age 1 on neurodevelopment using the Japanese version of the Ages and Stages Questionnaires, Third Edition (ASQ-3) [19]. In the previous study, we observed developmental delays in all five domains evaluated by this screening tool at age 1, with particularly significant delays in gross motor function in children who received general anesthesia [20]. However, only neurodevelopment at age 1 was evaluated. The JECS continues to conduct follow-ups twice a year as the child grows. In this study, we used the results obtained by the age of 4 years from children participating in the JECS to examine the effects of general anesthesia exposure in childhood on neurodevelopment in growing children.

## Methods

### Study design and population

The data used in this study were obtained from the JECS, which enrolled pregnant women from 15 regional centers nationwide between January 2011 and March 2014, as reported in a previous study [19]. The JECS is a birth cohort study that evaluates the impact of various environmental factors on the growth and development of children [20, 21]. The protocol of the JECS was reviewed and approved by the Ministry of the Environment’s Institutional Review Board on Epidemiological Studies (Ethical Number: No.100910001) and by the Ethics Committees of all participating institutions. Written informed consent was obtained from all participants in the study. In the present study, we used the “jecs-ta-20190930” and “jecs-qa-20210401” datasets, released in October 2019 and April 2021, respectively.

### Data collection

After study registration, each mother completed two self-administered questionnaires during the first and second or third trimester of pregnancy. The medical records transcriptions of mothers and children at the time of delivery were collected by the doctors, midwives, nurses, and research co-ordinators immediately after birth and 1 month post-partum. The mother/guardian was asked to complete a self-administered questionnaire on the child’s development every 6 months up to 4 years after the first 6 months of age, similar to our previous study. We used the Japanese translation of the Ages and Stages Questionnaires, Third Edition (j-ASQ-3), which is a parent-completed developmental screening tool designed for children from 1 to 66 months of age and comprises five domains: communication, gross motor, fine motor, problem solving, and personal–social) [22–24]. Normal development was defined as an equal or higher score for each domain than the cutoff scores adjusted for each age in Japanese children [25, 26], whereas neurodevelopmental delay was defined as scores that were lower than the cutoff. The presence or absence of exposure to general anesthesia was extracted from the “surgery under general anesthesia” item of the questionnaire for each time point. Notably, this question does not convey information about the date and time of surgery, type of anesthesia, diagnosis, or type of surgery.

### Statistical analysis

**Fig. 1:**
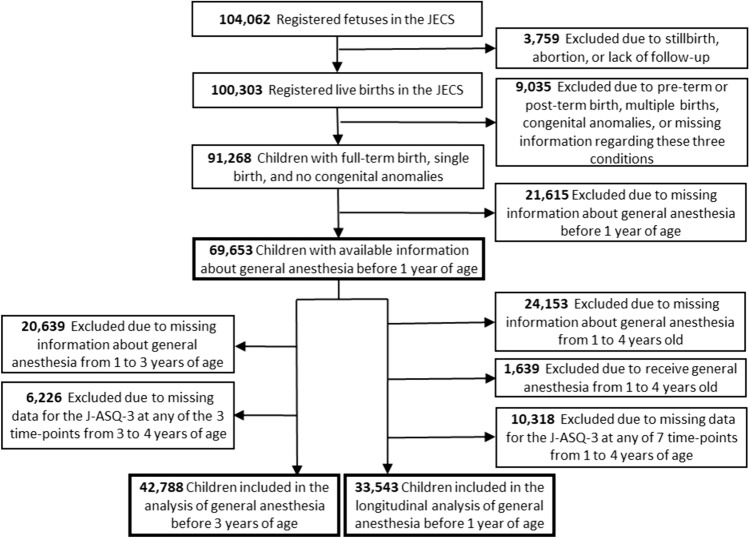
Flowchart of the study participant selection. *J-ASQ-3* Japanese translation of the Ages and Stages Questionnaire (Third Edition), *JECS* Japan Environment and Children’s Study

Of the 104,062 fetal records registered in the JECS, 100,303 were live births. We analyzed the effects of general anesthesia received by age 1 on neurodevelopment up to age 4 in 69,653 children born between 37 and 41 weeks of pregnancy via single-vaginal delivery, after excluding pre-term, post-term births, multiple births, children with congenital anomalies, and those who lacked information on the history of general anesthesia before age 1 (Fig. 1). Analysis was performed using multiple logistic regression, which was adjusted for the following covariates: maternal age at delivery, body mass index (BMI) before pregnancy, marital status, smoking status, early pregnancy occupational status, educational background, annual household income, fetal sex, gestational age at birth, use of any medication before and during pregnancy, mode of delivery, presence or absence of epidural analgesia, birth weight, presence or absence of nursery school attendance at 6 and 12 months of age, breastfeeding method during the first 6 months of life, and presence or absence of cohabiting siblings. The risk of neurodevelopmental delay in the five domains of the J-ASQ-3 every 6 months in children from 1 to 4 years of age was presented using adjusted odds ratios (aOR) and 95% confidence intervals (CIs). Furthermore, a subset of the cohort was also analyzed to exclude the effects of general anesthesia received after age 1. The risk of neurodevelopmental delay due to general anesthesia before age 1 was investigated in 33,543 children who were confirmed not to receive general anesthesia after age 1 with valid results from the J-ASQ-3 at all seven time points. The associations between general anesthesia before age 1 and the incidence of neurodevelopmental delay every 12 months after 1 year of age were also estimated. In these analyses, neurodevelopmental delay at 24 months in children without delay at 12 months indicated the incidence of delay during 12–24 months. Similarly, neurodevelopmental delay at 36 and 48 months was assessed in children without delay at 24 and 36 months, respectively. Therefore, children with preexisting neurodevelopmental delay before undergoing surgery were excluded. The aORs and 95% CI for the new onset of neurodevelopmental delay in the five domains of the J-ASQ-3 every 12 months from 1 to 4 years are shown.

Additionally, for 42,788 children with valid results from three J-ASQ-3 evaluations performed at 3–4 years of age for whom the history of general anesthesia before age 3 was available, the relationship between neurodevelopment at 36–48 months and age at first exposure to general anesthesia before age 3 was assessed.

Missing variables were treated using multiple imputation to reduce the effect of non-response bias due to missing covariates. Statistical analysis was performed using SPSS Statistics version 27 (IBM Corp, Armonk, NY) and a *p* value < 0.05 was considered significant.

## Results

### Participant characteristics

Table 1 presents the characteristics of both the mothers and children analyzed in this study. Of the 69,653 children for whom data on the presence or absence of general anesthesia exposure before age 1 was obtained, 570 (0.8%) received general anesthesia only once before age 1, and 98 (0.1%) received general anesthesia two or more times. Of the 33,543 children who did not receive general anesthesia between the ages of 1 and 4 years and completed the questionnaire evaluation seven times between 12 and 48 months, 159 (0.5%) received general anesthesia once before age 1 and 24 (0.1%) received it two or more times.

**Table 1:** Characteristics of the study participants

	Participants with available information about general anesthesia before 1 year of age (*n* = 69,653)	Participants included in the longitudinal analysis of general anesthesia before 1 year of age (*n* = 33,543)
Maternal characteristics		
Age at delivery, years		
Mean (SD)	31.3 (4.9)	31.6 (4.8)
< 20	906 (1.3)	258 (0.8)
20–29	24,301 (34.9)	11,119 (33.1)
30–39	41,363 (59.4)	20,577 (61.3)
≥ 40	3,080 (4.4)	1,589 (4.7)
Missing	3 (0.0)	0 (0.0)
Gestational age at birth, weeks, mean (SD)	39.5 (1.1)	39.5 (1.1)
Mode of delivery		
Spontaneous	40,789 (58.6)	19,560 (58.3)
Induced	12,612 (18.1)	6,194 (18.5)
Vacuum or forceps	4,150 (6.0)	2,022 (6.0)
Cesarean section	11,927 (17.1)	5,680 (16.9)
Missing	175 (0.3)	87 (0.3)
Epidural analgesia		
Yes	1,461 (2.1)	743 (2.2)
No	68,192 (97.9)	32,800 (97.3)
Use of any medication before pregnancy		
Yes	59,471 (85.4)	28,993 (86.3)
No	8,916 (12.8)	4,047 (12.1)
Missing	1,266 (1.8)	563 (1.7)
Use of any medication during pregnancy		
Yes	47,775 (68.6)	23,432 (69.9)
No	20,650 (29.6)	9,623 (28.7)
Missing	1,228 (1.8)	488 (1.5)
Body mass index before pregnancy		
Mean (SD)	21.2 (3.2)	21.0 (3.1)
< 18.5	11,159 (16.0)	5,469 (16.3)
18.5–24.9	51,555 (74.0)	25,034 (74.6)
≥ 25.0	6,893 (9.9)	3,022 (9.0)
Missing	46 (0.1)	18 (0.1)
Marital status during the first trimester		
Married (including common-law marriage)	66,380 (95.3)	32,238 (96.1)
Divorced	2,199 (3.2)	963 (2.9)
Widowed	8 (0.0)	5 (0.0)
Other	440 (0.6)	159 (0.5)
Missing	626 (0.9)	178 (0.5)
Occupation during the first trimester		
Yes	45,357 (65.1)	21,786 (64.9)
No	22,273 (32.0)	11,022 (32.9)
Missing	2,023 (2.9)	735 (2.2)
Smoking status		
Never smoker	41,784 (60.0)	21,542 (64.2)
Ex-smoker (quit before pregnancy)	16,198 (23.3)	7,599 (22.7)
Ex-smoker (quit during early pregnancy)	8,260 (11.9)	3,302 (9.8)
Current smoker	2,602 (3.7)	856 (2.6)
Missing	809 (1.2)	249 (0.7)
Alcohol consumption		
Never drinker	23,761 (34.1)	11,762 (35.1)
Ex-drinker	38,174 (54.8)	18,233 (54.4)
Current drinker	7,063 (10.1)	3,361 (10.0)
Missing	655 (0.9)	187 (0.6)
Education, years		
≤ 12	22,851 (32.8)	9,867 (29.4)
13–15	29,501 (42.4)	14,356 (42.8)
≥ 16	16,576 (23.8)	9,133 (27.2)
Missing	725 (1.0)	187 (0.6)
Annual household income		
< 4,000,000 JPY	24,774 (35.6)	11,261 (33.6)
< 8,000,000 JPY	32,729 (47.0)	16,547 (49.3)
≥ 8,000,000 JPY	7,430 (10.7)	3,852 (11.5)
Missing	4,720 (6.8)	1,883 (5.6)
Child characteristics		
Sex		
Male	35,433 (50.9)	16,959 (50.6)
Female	34,220 (49.1)	16,584 (49.4)
Birth weight, grams		
Mean (SD)	3,064.5 (364.5)	3,062.7 (362.7)
< 2500	3,608 (5.2)	1,729 (5.2)
2500–3999	65,396 (93.9)	31,508 (93.9)
≥ 4000	616 (0.9)	290 (0.9)
Missing	34 (0.0)	16 (0.0)
Feeding method for the first 6 months of life		
Breast	30,523 (43.8)	15,056 (44.9)
Bottle	6,373 (9.1)	2,553 (7.6)
Mixed	32,757 (47.0)	15,934 (47.5)
Older children living together		
Yes	37,707 (54.1)	17,223 (51.3)
No	31,551 (45.3)	16,219 (48.4)
Missing	395 (0.6)	101 (0.3)
Nursery attendance at 12 months of age		
Yes	19,175 (27.5)	8,160 (24.3)
No	50,214 (72.1)	25,309 (75.5)
Missing	264 (0.4)	74 (0.2)
General anesthesia before 1 year of age		
None	68,985 (99.0)	33,360 (99.5)
Once	570 (0.8)	159 (0.5)
≥ 2 times	98 (0.1)	24 (0.1)
General anesthesia between 1 and 2 years of age		
None	57,601 (82.7)	33,543 (100.0)
Once	809 (1.2)	–
≥ 2 times	102 (0.1)	–
Missing	11,141 (16.0)	–
General anesthesia between 2 and 3 years of age		
None	53,119 (76.3)	33,543 (100.0)
Once	668 (1.0)	–
≥ 2 times	196 (0.3)	–
Missing	15,670 (22.5)	–
General anesthesia between 3 and 4 years of age		
None	57,081 (82.0)	33,543 (100.0)
Once	854 (1.2)	–
≥ 2 times	119 (0.2)	–
Missing	11,599 (16.7)	–

Data are *n* (%) unless otherwise specified

*SD* standard deviation, *JPY* Japanese yen

### Relationship between general anesthesia before 1 year of age and neurodevelopment up to 4 years of age

Table 2 shows the numbers and percentage of children with scores below the cutoff value for each questionnaire domain every 6 months during the 12 to 48-month period by the frequency of general anesthesia received by age 1. Throughout the entire study period, the proportion of children with neurodevelopmental delay in all five domains was the lowest among those who had never received general anesthesia, followed by those who received it only once and those who received it two or more times.

**Table 2:** Neurodevelopmental delay in each domain of the Japanese translation of the Ages and Stages Questionnaire (Third Edition) in children, according to the cumulative number of general anesthesia exposure before 1 year of age

	None *n* (%)	Once *n* (%)	≥ 2 times *n* (%)
12 months			
Subjects (*n* = 63,273)	62,681	505	87
Communication	66 (0.1)	1 (0.2)	3 (3.4)
Gross motor	3,399 (5.4)	64 (12.7)	25 (28.7)
Fine motor	3,531 (5.6)	46 (9.1)	20 (23.0)
Problem solving	3,126 (5.0)	44 (8.7)	18 (20.7)
Personal–social	690 (1.1)	16 (3.2)	3 (3.4)
18 months			
Subjects (*n* = 58,731)	58,191	458	82
Communication	1,198 (2.1)	18 (3.9)	7 (8.5)
Gross motor	2,539 (4.4)	61 (13.3)	17 (20.7)
Fine motor	2,362 (4.1)	39 (8.5)	9 (11.0)
Problem solving	2,205 (3.8)	40 (8.7)	9 (11.0)
Personal–social	1,369 (2.4)	28 (6.1)	12 (14.6)
24 months			
Subjects (*n* = 60,131)	59,580	474	77
Communication	2,175 (3.7)	32 (6.8)	10 (13.0)
Gross motor	3,257 (5.5)	54 (11.4)	19 (24.7)
Fine motor	1,153 (1.9)	25 (5.3)	8 (10.4)
Problem-solving	2,344 (3.9)	37 (7.8)	9 (11.7)
Personal–social	1,536 (2.6)	36 (7.6)	8 (10.4)
30 months			
Subjects (*n* = 58,429)	57,888	459	82
Communication	2,598 (4.5)	44 (9.6)	12 (14.6)
Gross motor	2,330 (4.0)	39 (8.5)	18 (22.0)
Fine motor	3,166 (5.5)	47 (10.2)	11 (13.4)
Problem solving	3,114 (5.4)	42 (9.2)	14 (17.1)
Personal–social	1,772 (3.1)	38 (8.3)	10 (12.2)
36 months			
Subjects (*n* = 59,401)	58,581	469	81
Communication	2,090 (3.6)	41 (8.7)	10 (12.3)
Gross motor	2,405 (4.1)	39 (8.3)	16 (19.8)
Fine motor	4,130 (7.0)	54 (11.5)	16 (19.8)
Problem solving	4,054 (6.9)	54 (11.5)	10 (12.3)
Personal–social	1,718 (2.9)	33 (7.0)	11 (13.6)
42 months			
Subjects (*n* = 58,040)	57,512	490	78
Communication	1,500 (2.6)	28 (6.2)	9 (11.5)
Gross motor	2,328 (4.0)	43 (9.6)	15 (19.2)
Fine motor	2,785 (4.8)	42 (9.3)	9 (11.5)
Problem solving	3,044 (5.3)	43 (9.6)	13 (16.7)
Personal–social	2,353 (4.1)	42 (9.3)	12 (15.4)
48 months			
Subjects (*n* = 56,270)	55,756	439	75
Communication	2,367 (4.2)	41 (9.3)	8 (10.7)
Gross motor	2,980 (5.3)	57 (13.0)	15 (20.0)
Fine motor	3,494 (6.3)	46 (10.5)	19 (25.3)
Problem solving	1,806 (3.2)	38 (8.7)	8 (10.7)
Personal–social	2,916 (5.2)	42 (9.6)	11 (14.7)

**Fig. 2:**
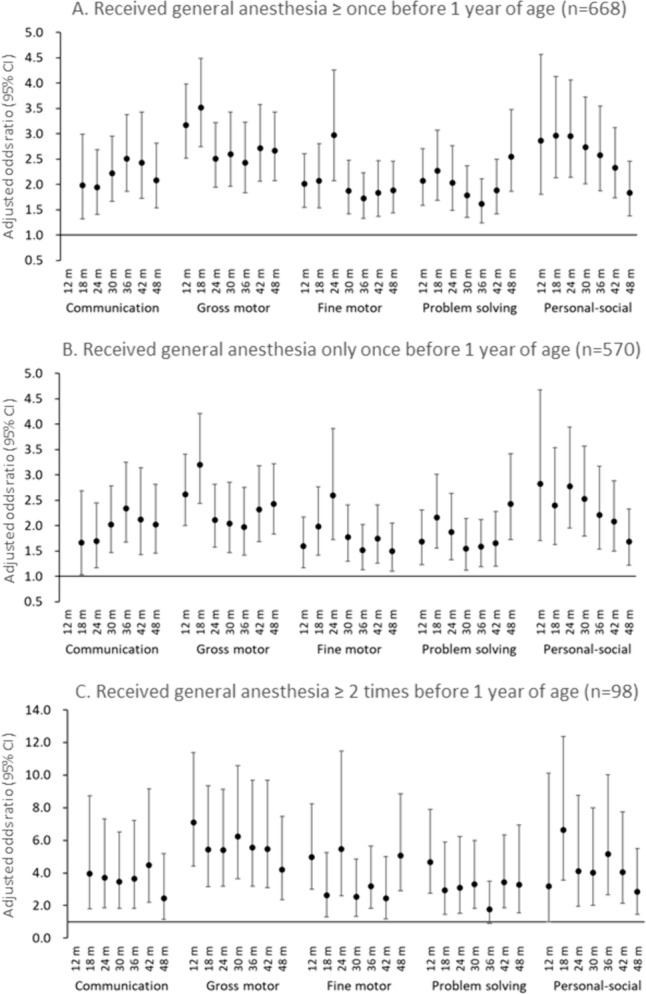
Association of general anesthesia ≥ once (**A**), only once (**B**), and ≥ 2 times (**C**) before 1 year of age with neurodevelopmental delay in children (*n* = 69,653). Adjusted odds ratios and 95% confidence intervals (CIs) for neurodevelopmental delay from the age of 12 to 48 months in each domain of the Japanese translation of the Ages and Stages Questionnaire (Third Edition) versus children who did not receive general anesthesia before age 1. Adjusted for maternal age at delivery, body mass index before pregnancy, marital status, smoking status, early pregnancy occupational status, educational background, annual household income, fetal sex, gestational age at birth, use of any medication before and during pregnancy, mode of delivery, presence or absence of epidural analgesia, birth weight, presence or absence of nursery school attendance at 6 and 12 months of age, breastfeeding method during the first 6 months of life, and presence or absence of cohabiting siblings. A multiple imputation method was used to reduce potential selection bias from missing variables. For communication delay at 12 months, the adjusted odds ratio could not be calculated due to the small number of participants with a score below the cutoff value

Figure 2 shows the results of logistic regression analyses of the relationship between general anesthesia before age 1 and the 6-monthly questionnaire performed between 12 and 48 months for 69,653 participants with available information on the presence or absence of general anesthesia exposure before age 1. Of the participants, 668 children received general anesthesia at least once by the age of 1 year, and they had significantly higher aORs for developmental delay in all five domains over the entire 12- to 48-month study period than those who did not receive general anesthesia before age 1. The largest aOR was 3.51 (95% CI 2.75–4.49) for gross motor delay at 18 months of age. In the personal–social domain, the aOR for developmental delay at 18 months was the highest at 2.97 (95% CI 2.13–4.13) and also significant at 1.84 (95% CI 1.37–2.45) at 48 months.

**Fig. 3:**
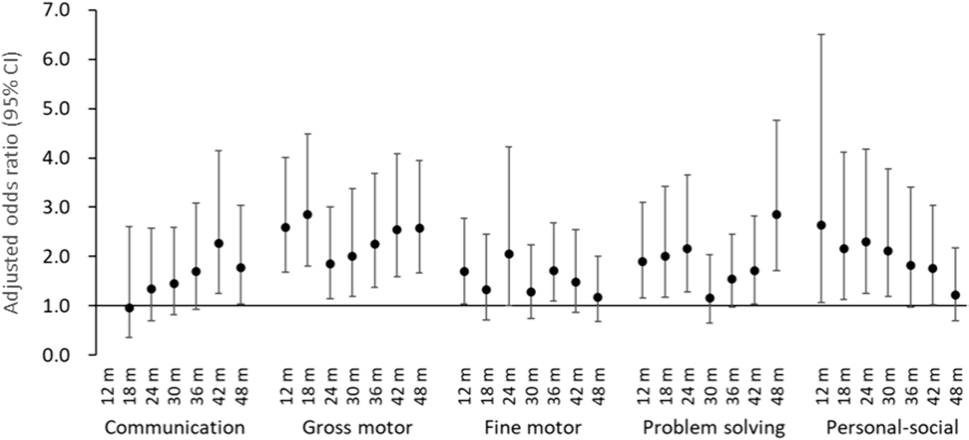
Association of general anesthesia before 1 year of age with neurodevelopmental delay in children who did not receive general anesthesia during 1–4 years of age (*n* = 33,543). Adjusted odds ratios and 95% confidence intervals (CIs) for neurodevelopmental delay from the age of 12 to 48 months in each domain of the Japanese translation of the Ages and Stages Questionnaire (Third Edition) versus children who did not receive general anesthesia before age 1. Adjusted for maternal age at delivery, body mass index before pregnancy, marital status, smoking status, early pregnancy occupational status, educational background, annual household income, fetal sex, gestational age at birth, use of any medication before and during pregnancy, mode of delivery, presence or absence of epidural analgesia, birth weight, presence or absence of nursery school attendance at 6 and 12 months of age, breastfeeding method during the first 6 months of life, and presence or absence of cohabiting siblings. A multiple imputation method was used to reduce potential selection bias from missing variables. For communication delay at 12 months, the adjusted odds ratio could not be calculated due to the small number of participants with a score below the cutoff value

Figure 3 shows the analysis results of 33,543 participants who did not receive general anesthesia during the age of 1–4 years and all questionnaire results during the period were validated to exclude the effects of general anesthesia received after age 1. Of the participants, 183 children received general anesthesia before age 1. Except for communication at 18 months, the aORs for developmental delay in all domains were greater than 1 over the entire period, compared with those who did not receive general anesthesia before age 1. The aOR for the gross motor delay was significant throughout the entire period, whereas other domains were not consistently significant at all time points. For example, the aORs for developmental delay in the fine motor and personal–social domains were not significant at 48 months.

**Fig. 4:**
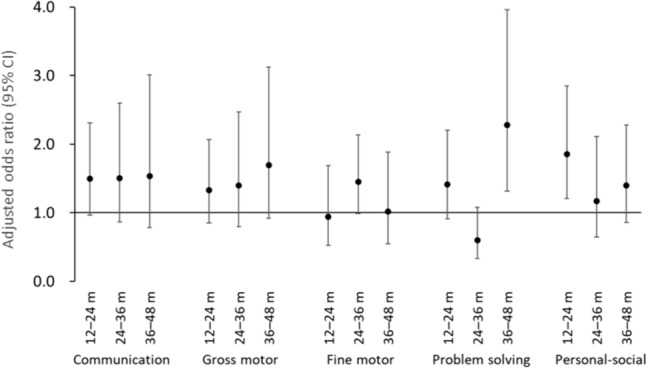
Association of general anesthesia before 1 year of age with the incidence of neurodevelopmental delay in children who did not receive general anesthesia during 1–4 years of age (*n* = 33,543). Adjusted odds ratios and 95% confidence intervals (CIs) for the incidence of neurodevelopmental delay every 12 months after 1 year of age in each domain of the Japanese translation of the Ages and Stages Questionnaire (Third Edition) versus children who did not receive general anesthesia before 1 year of age. Adjusted for maternal age at delivery, body mass index before pregnancy, marital status, smoking status, early pregnancy occupational status, educational background, annual household income, fetal sex, gestational age at birth, use of any medication before and during pregnancy, mode of delivery, presence or absence of epidural analgesia, birth weight, presence or absence of nursery school attendance at 6 and 12 months of age, breastfeeding method during the first 6 months of life, and presence or absence of cohabiting siblings. A multiple imputation method was used to reduce potential selection bias from missing variables

Figure 4 shows the association between general anesthesia before age 1 and the incidence of neurodevelopmental delay every 12 months after 1 year of age. The aORs for the incidence of developmental delay in problem solving and personal–social were significant during 36–48 months and 12–24 months, respectively. However, the other risks for the incidence of neurodevelopmental delay were not observed.

### Relationship between age at first general anesthesia administration and neurodevelopment at 3–4 years of age

**Fig. 5:**
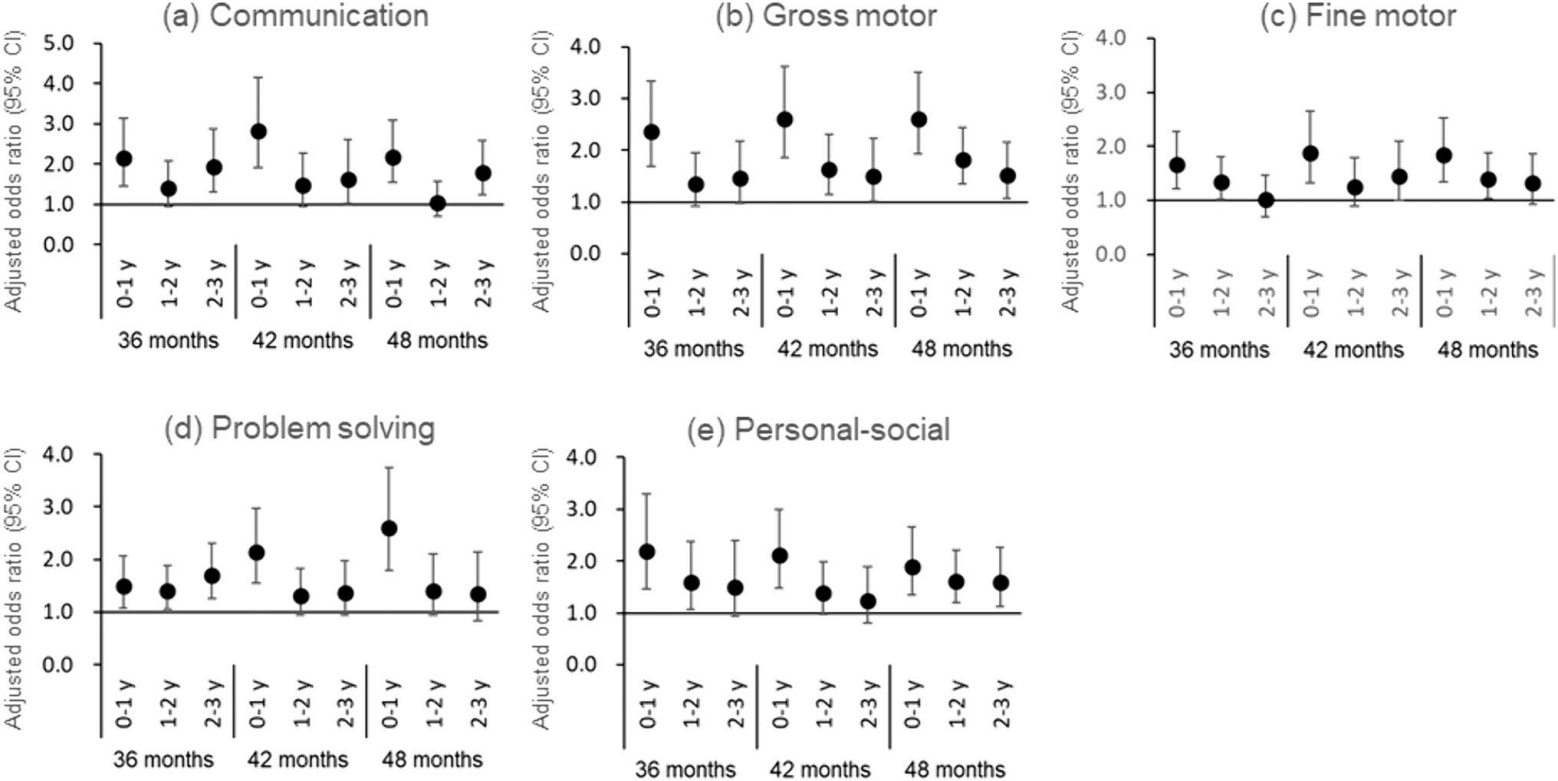
Association of general anesthesia with neurodevelopmental delay at 36–48 months and the age at first exposure to receiving general anesthesia before 3 years of age (*n* = 42,788) Adjusted odds ratios and 95% confidence intervals (CIs) for neurodevelopmental delay from age 36 to 48 months in each domain of the Japanese translation of the Ages and Stages Questionnaire (Third Edition). Adjusted for maternal age at delivery, body mass index before pregnancy, marital status, smoking status, early pregnancy occupational status, educational background, annual household income, fetal sex, gestational age at birth, use of any medication before and during pregnancy, mode of delivery, presence or absence of epidural analgesia, birth weight, presence or absence of nursery school attendance at 6 and 12 months of age, breastfeeding method during the first 6 months of life, and presence or absence of cohabiting siblings. A multiple imputation method was used to reduce potential selection bias from missing variables

Figure 5 depicts the results of logistic regression analyses of the relationship between neurodevelopmental delay at 36–48 months and age at first exposure to general anesthesia before age 3. Of 42,788 participants included in this analysis, 471, 589, and 415 children received their first general anesthesia before 1 year, at the ages of 1–2 and 2–3 years, respectively. Children who received general anesthesia before age 1 had significantly high aORs for developmental delay in all five domains throughout the 36- to 48-month study period. Children who received general anesthesia for the first time at the ages of 1–2 or 2–3 years had a small risk of developmental delay in all domains, and the delay in the problem solving domain was not observed at 42 or 48 months. Moreover, the aORs for communication delay in children who received general anesthesia at 1–2 years of age and fine motor skill development delay for those who received general anesthesia at 2–3 years were not significant at any time point.

## Discussion

How general anesthesia exposure at an early age affects the psychosomatic development of growing children remains inconclusive. Several retrospective studies have examined the association between childhood exposure to general anesthesia and neurodevelopmental deficit, with some studies indicating a significant association [27–33] and others not finding any link [34–40]. However, most studies that reported that general anesthesia does not affect neurodevelopment evaluated children shortly after surgery [35] or during adolescence [36–38]. Previously, we examined the association between the number of surgeries performed under general anesthesia before age 1 and neurodevelopment in babies at age 1, using the JECS data, and found that the higher the frequency of general anesthesia exposure, the higher is the proportion of neurodevelopmental delay according to the J-ASQ-3 [19]. We reported that children who underwent general anesthesia three or more times by age 1 had a significant developmental delay in all five domains of the questionnaire compared to children who did not receive general anesthesia. However, we were unable to exclude the possibility that a higher frequency of exposure to general anesthesia due to the severity of the underlying diagnosis influenced developmental delay. Moreover, further evaluation of the impact of general anesthesia on the development of children beyond age 1 was necessary. In this study, we evaluated neurodevelopment up to age 4.

The present study found that children who received general anesthesia by age 1 faced a higher risk of developmental delay in all five domains of the J-ASQ-3 throughout the 12- to 48-month follow-up period than those who had never received general anesthesia. In a subset of 33,543 children who did not receive general anesthesia after age 1, the delay in the development of gross motor skills was significant throughout the entire period; however, the risks of developmental delay in the fine motor and personal–social domains were not significant at 48 months. Additionally, the association between general anesthesia before 1 year of age and the incidence of neurodevelopmental delay was not observed beyond age 1, except for personal–social delay during 12–24 months and problem solving delay during 36–48 months. These findings suggest that the effect of general anesthesia on neurodevelopmental delay in children was little after age 1. We believe that the decrease in the risk of developmental delay in personal–social skills with age is due to the support system for children with developmental disabilities found by health examinations during infancy. Moreover, the social skills of infants and young children generally advance with age and are acquired through interactions with others; thus, these deficits may have been attenuated by growth. The risk of the incidence of problem solving delay was observed during 36–48 months. Recently, the adverse effects of early-life exposure to anesthesia on neurocognitive function have been observed in mice [41]. Since thinking ability is required for problem solving, we suggest that anesthetics may affect the development of the immature brain. Thus, it is necessary to track the effects of neurodevelopment on children’s growth over a longer period of time due to the inter-individual differences in early childhood development, irrespective of general anesthesia administration.

Analysis of age at the time of first exposure to general anesthesia revealed that the risk of neurodevelopmental delay in all domains was present at age 4 when general anesthesia was received before age 1. However, the risk of developmental delay decreased when exposure occurred for the first time at age 1 or beyond and was not significant in some domains. Thus, our results suggest elective surgery is desirable to be performed after age 1.

The strength of our study was that we used data from 69,653 children enrolled in a large birth cohort study; thus, we were able to evaluate the longitudinal relationship between general anesthesia received in childhood and neurodevelopment at intervals of 6 months thereafter, owing to its prospective cohort design. Moreover, we were able to accurately evaluate neurodevelopment specific to the current situation in Japan by using the J-ASQ-3 to evaluate neurodevelopment in children using cut-ff values for each month of age for Japanese children.

This study also had several limitations. First, the type and duration of anesthetic exposure were unknown because information relating to the surgical procedure for general anesthesia was obtained through a parent/guardian-administered questionnaire. Also, it may lead to a misclassification, as some children might have taken particular anesthetics to undergo some procedures or examinations, and these children may be misclassified into the group without general anesthesia. This limitation is due to the nature of an observational study. In previous population-based studies on the effects of anesthesia, the information has been collected from questionnaire [42] or administrative data [43] other than medical records [15, 16]. The information from the questionnaire may differ from medical records and should be used with caution. It is desirable to review the medical records of the children who were reported to receive general anesthesia. in a questionnaire by parents. We stated that it is desirable to review the medical records of the children who were reported to receive general anesthesia in a questionnaire by parents. The protocol of the JECS. Many information of the participants has been obtained from questionnaires [20, 21, 44]. To validate the information about general anesthesia from the questionnaire, a review of medical records should be considered in the future. Several studies have reported that anesthesia during surgical procedures may increase the risk of postoperative adverse events in infants and young children [27–33]. A retrospective cohort study reported the association between major surgery in infancy and neurodevelopmental impairment, but the authors considered that the role of general anesthesia remained unproven because data on the type and duration of anesthesia were unavailable [45]. Because we were unable to evaluate the exposure time of general anesthesia in this study, the observed risk may be overestimated. Second, we were also unable to evaluate the diagnosis of diseases that required general anesthesia. If a surgical procedure under general anesthesia is required in childhood, the pathology and severity of the causative disease may impact neurodevelopmental delay. According to the National Clinical Database (NCD), the number of inguinal hernia was highest among surgical diseases under 1 year in Japan during this study period, followed by anorectal malformation and intestinal atresia [46, 47]. In this study, children with congenital anomalies, such as anorectal malformation and intestinal atresia, were excluded from this analysis. Therefore, the total number of children who received general anesthesia by age 1 was 1419 among the overall participants in the JECS (data not shown); however, the number of children considered to have relatively mild diseases, such as inguinal hernia, included in this analysis was limited to 668. However, the possible effect of the underlying disease on neurodevelopment cannot be ruled out. Third, developmental delay was evaluated using a questionnaire and not based on a physician’s diagnosis. Mothers of children who received general anesthesia may be more aware of their child’s neurodevelopmental delay. However, the ASQ-3 is a widely accepted tool for assessing neurodevelopment in children [22–26]. Finally, this study evaluated the effects of general anesthesia administration in early childhood on neurodevelopment up to the age of 4 years. Therefore, many children of the participants in the JECS were excluded from the analyses because of unavailable data on neurodevelopment at any time point. The possibility of a non-responsive bias due to the neurodevelopment of the children should also be considered. Further investigation of the long-term effects on neurodevelopment with age is required. Since the JECS is ongoing and will continue until the children reach age 13, the impact of general anesthesia exposure in early childhood on neurodevelopment in older children can also be examined in the future.

## Conclusions

The effects of general anesthesia administered in early childhood on neurodevelopment up to age 4 were examined using data from a large birth cohort study. Children who received general anesthesia by age 1 had neurodevelopmental delays in all five domains of the J-ASQ-3 at age 4. The largest risk was for gross motor delay at 18 months. Conversely, the effects on the incidence of neurodevelopmental delays after age 3 were not observed except for problem solving at 48 months. Children who received general anesthesia for the first time after the age of 1 year also faced a risk of neurodevelopmental delay, but the effect was considerably small. These results indicate that the administration of general anesthesia at a young age may affect neurodevelopment in children, but the effect may be small after 1 year of age. Given the reduced impact of general anesthesia received after age 1, more favorable neurodevelopmental outcomes are expected if elective surgical procedures are performed after age 1.

## Data Availability

Data are unsuitable for public deposition due to ethical restrictions and legal framework of Japan. It is prohibited by the Act on the Protection of Personal Information (Act No. 57 of 30 May 2003, amendment on 9 September 2015) to publicly deposit the data containing personal information. Ethical Guidelines for Medical and Health Research Involving Human Subjects enforced by the Japan Ministry of Education, Culture, Sports, Science and Technology and the Ministry of Health, Labour and Welfare also restricts the open sharing of the epidemiologic data. All inquiries about access to data should be sent to: jecs-en@nies.go.jp. The person responsible for handling enquiries sent to this e-mail address is Dr Shoji F. Nakayama, JECS Programme Office, National Institute for Environmental Studies.

## Abbreviations

aOR: Adjusted odds ratio
ASQ-3: Ages and Stages Questionnaires, Third Edition
BMI: Body mass index
CI: Confidence interval
FDA: U.S. Food and Drug Administration
GAS: General Anaesthesia compared to Spinal Anesthesia
J-ASQ-3: Japanese translation of the Ages and Stages Questionnaires, Third Edition
JECS: Japan Environment and Children’s Study
MASK: Mayo Anesthesia Safety in Kids
N_2_O: Nitrous oxide
NCD: National Clinical Database
PANDA: Pediatric Anesthesia Neurodevelopment Assessment
